# The Use of Artificial Intelligence in Medical Imaging: A Nationwide Pilot Survey of Trainees in Saudi Arabia

**DOI:** 10.3390/clinpract12060090

**Published:** 2022-10-28

**Authors:** Ahmad A. Mirza, Omar M. Wazgar, Ammar A. Almaghrabi, Roaa M. Ghandour, Sarah A. Alenizi, Abdulrahim A. Mirza, Khalid S. Alraddadi, Fayzah H. Al-Adwani, Mohammed A. Alsakkaf, Sattam M. Aljuaid

**Affiliations:** 1 Department of Otolaryngology–Head and Neck Surgery, Faculty of Medicine in Rabigh, King Abdulaziz University, Jeddah 21589, Saudi Arabia; 2 Department of Otolaryngology–Head and Neck Surgery, Temerty Faculty of Medicine, University of Toronto, Toronto, ON M5S 3H2, Canada; 3 Department of Radiology, Al-Hada Armed Forces Hospital, Taif 26792, Saudi Arabia; 4 Department of Radiology, Al-Noor Specialist Hospital, Makkah 24241, Saudi Arabia; 5 Department of Radiology, King Abdullah Medical Complex, Jeddah 23816, Saudi Arabia; 6 Department of Radiology, King Fahad Armed Forces Hospital, Jeddah 23311, Saudi Arabia; 7 Department of Surgery—Division of Urology, King Abdulaziz Medical City, Ministry of National Guard Health Affairs, Jeddah 21423, Saudi Arabia; 8 Department of Primary Health Care, Ministry of National Guard Health Affairs, King Saud Bin Abdul-Aziz University for Health Sciences, Jeddah 21423, Saudi Arabia; 9 Department of Surgery, Security Forces Hospital Program, Makkah 24251, Saudi Arabia; 10 Department of Otolaryngology–Head and Neck Surgery, Prince Mansour Military Hospital, Taif 26526, Saudi Arabia

**Keywords:** artificial intelligence, diagnostic imaging, education, radiology, Saudi Arabia

## Abstract

Artificial intelligence is dramatically transforming medical imaging. In Saudi Arabia, there are a lack of studies assessing the level of artificial intelligence use and reliably determining the perceived impact of artificial intelligence on the radiology workflow and the profession. We assessed the levels of artificial intelligence use among radiology trainees and correlated the perceived impact of artificial intelligence on the workflow and profession with the behavioral intention to use artificial intelligence. This cross-sectional study enrolled radiology trainees from Saudi Arabia, and a 5-part-structured questionnaire was disseminated. The items concerning the perceived impact of artificial intelligence on the radiology workflow conformed to the six-step standard workflow in radiology, which includes ordering and scheduling, protocoling and acquisition, image interpretation, reporting, communication, and billing. We included 98 participants. Few used artificial intelligence in routine practice (7%). The perceived impact of artificial intelligence on the radiology workflow was at a considerable level in all radiology workflow steps (range, 3.64–3.97 out of 5). Behavioral intention to use artificial intelligence was linearly correlated with the perceptions of its impact on the radiology workflow and on the profession (*p* < 0.001). Artificial intelligence is used at a low level in radiology. The perceived impact of artificial intelligence on radiology workflow and the profession is correlated to an increase in behavioral intention to use artificial intelligence. Thus, increasing awareness about the positive impact of artificial intelligence can improve its adoption.

## 1. Introduction

Artificial intelligence (AI) represents a revolution in data science and information technology because it improves automation tasking technology. AI refers to the simulation of human intelligence in digital-based systems, in which tasks requiring human intelligence can be performed without human intelligent inputs. The main subsets of AI are represented by machine learning (ML) and deep learning (DL) [1,2,3]. Many industries and domains are gradually becoming involved in AI. In the healthcare industry, AI is used on many levels including for diagnostics, therapeutic and surgical assistance, and record-keeping [4,5,6]. Medical imaging is a specialty that has likely benefited greatly from recent AI-based innovations and advances. The diagnostic technicality of medical imaging relies on different factors, of which the data acquisition and interpretation with minimum error are important elements; these are two remarkable functions of AI [7,8].

Several surveys have been conducted to examine radiology practitioners’ perceptions and use of AI technology [9,10,11,12,13,14,15,16]. Many clinicians agreed that AI has a positive impact on their profession. A survey of trainees of the Royal College of Physicians and Surgeons in Canada showed that 72% of respondents perceived AI as having a positive impact on workflow and/or clinical practice and patient experience [17]. However, AI has not been widely adopted in the radiology field. In the United States, as many as 38% of radiology trainees use AI in their practice [18]. In addition to the growing interest in, and applications of, AI in medical imaging, anxiety is increasing among radiologists about the potentially disrupting effect on radiology practice. A considerable number of radiology personnel (42%) concerns that AI will reduce medical imaging jobs [19,20]. Thus, it is imperative to understand the current beliefs and intended behavior of radiology professionals towards the AI integration into medical imaging in order to describe future needs for successful implementation.

In Saudi Arabia, the levels of knowledge, experience, and use of AI have been investigated among radiologists. However, most studies lack a reliable tool measuring broader dimensions of the level of AI use and perceived impact on the workflow and radiology profession [11,13,20]. We hypothesized that the trainees in our residency program have an improper knowledge about the role of AI in the radiology workflow and profession as there is no formal education on this subject. In the present study, we aimed at assessing the levels of exposure to AI radiology, including familiarity, experience, and level of current use. We also aimed to explore the perceived contributions of AI radiology in the workflow and radiology profession. Further, we assessed the levels of perceived ease of use (PEoU) and behavioral intention (BI) to AI use in routine radiology practice and explored their predictive factors.

## 2. Materials and Methods

### 2.1. Study Design and Participants

This cross-sectional study was performed in Saudi Arabia among all radiology residency trainees (R1s to R4s). The radiology program in Saudi Arabia is approved by the Saudi Commission for Health Specialties. It is a 4-year structured program with 2 years in each of 2 phases: junior and senior phase. The total number of trainees in the program was estimated as 585. The curriculum of radiology in Saudi Arabia includes courses and workshops covering radiology basics. However, there is no formal subject related to AI.

The study commenced after ethical approval was obtained from the scientific research center at the health services department of Armed Forces Hospital in Taif, Saudi Arabia (Approval Ref: 2021-06-577; Date of approval: 23 June 2021). The study was conducted in accordance with the Declaration of Helsinki principles. Participation was voluntary and anonymous.

### 2.2. Sampling Technique and Sample Size Determination

A convenience sampling was used to approach all trainees (*N* = 585). Sample size (*n* = 233) was calculated, by using (Raosoft, Inc., Seattle, WA, USA), to detect an unknown proportion (*p* = 50%) of participants who have a previous experience in AI radiology, with 80% statistical power, 95% confidence interval (95% CI), and 0.05 type I error.

### 2.3. Questionnaire and Data Collection

A structured questionnaire was designed by the author, aided by a targeted non-systematic non-strategic literature search using search words: “artificial intelligence” AND “radiology”. The questionnaire comprised five parts, which are described below.

Part A: collected participants’ demographic and professional data such as age, gender, sector (Ministry of Health, University, Military, and Other), and academic degree.

Part B: assessed the exposure to AI using three subscales: (1) self-assessed knowledge level about AI, ML, DL, and data science (four items); (2) levels of involvement and interest in AI (two items); and (3) current level of use (LoU) of AI (Appendix A) using an adaptation of the LoU dimension scale from the Concerns-based Adoption Model (CBAM), which evaluates human factors that may interfere with successful implementation of an innovation [21]. The LoU was designed as an eight-level scale ranging from level 0 (no experience and no significant knowledge or active interest in being involved) to level 6 (engaged use with critical view regarding the functionality and improvement possibilities of the system).

Part C: explored perceptions about AI opportunities and applications in radiology, using a five-point Likert-type scale that measured the perceived level of impact (from 1 (no impact) to 5 (drastic impact)) on 10 dimensions of the standard radiology workflow (Appendix B). Items in this part were developed conforming to the six-step standard workflow in radiology, which includes ordering and scheduling, protocoling, and acquisition, image interpretation, reporting, communication, and billing [8].

Part D: explored attitudes regarding the impact of AI on the radiology profession. A five-point Likert-type scale was developed to measure the perceived impact of AI implementation (from −2 (very negative impact) to +2 (very positive impact)) on ten dimensions of the radiology profession such as ethics, income, job opportunities, and role in society (Appendix C).

Part E: explored the PEoU and BI to AI use in radiology (Appendix D) using a six-item scale (three items for PEoU and three items for BI) that was developed based on the Technology Acceptance Model (TAM) by Davis, Bagozzi, and Warshaw (1989), which originally aimed to provide an explicative and predictive model of people’s readiness for and willingness to adopt a novel technology [22,23].

The questionnaire was validated by assessing the face and content validity of Parts B–E and by analyzing the internal consistency of all the Likert-type scales. The questionnaire was edited for online use on Google Forms. The link was disseminated to trainees’ groups/networks. The survey link was kept open for 21 days in July 2021, during which two reminders, with a time interval of 7 days, were sent to prompt participation.

### 2.4. Statistical Analysis

Scores were calculated to reflect the study outcomes including knowledge level, practice level, LoU, level of perceived impact on standard radiology workflow, perceived impact on radiologist profession, PEoU, and BI. The concerned variables were analyzed as numerical or categorical variables depending on their linearity and distribution. Statistical analysis was performed using SPSS version 21.0 for Windows (SPSS Inc., Chicago, IL, USA). Categorical variables were presented as the frequency and percentage, while numerical variables were presented as the mean ± standard deviation (SD). Inferential analysis was performed to analyze the different associations that were stated in the objectives using appropriate tests. Where applicable, continuous data were compared using independent *t*-test or one-way analysis of variance (ANOVA). The correlation between scores was determined using linear regression and Person’s correlation. Independent factors for PEoU and BI were assessed using stepwise linear regression with entry *p*-value of 0.05 and removal *p*-value of 0.10 for variable selection. A *p*-value of <0.05 was considered to represent statistical significance.

## 3. Results

### 3.1. Participants’ Characteristics

A total of 98 radiology residents responded to the current survey (an overall response rate of 17%); 51 of them were male, and their mean (SD) age was 27.59 (2.02) years. Makkah Province was predominantly represented (57% of the participants), followed by Riyadh (17.5%) and the Eastern Province (16.5%). Regarding professional characteristics, the typical participant had a bachelor’s degree (96%), was working at an institution affiliated with the Ministry of Health (79%), and was involved in a mixed academic/non-academic activity (61%). Detailed characteristics are presented in Table 1.

### 3.2. Exposure to and Interest in Artificial Intelligence in Radiology

Overall, 45% of the participants indicated that they were familiar with AI radiology. Relatively lower levels of familiarity were observed for the other concepts including ML, DL, and data science. On the other hand, the majority of participants indicated that they were involved or interested in AI (86%). Table 2 demonstrates participants’ familiarity and interest in AI.

### 3.3. Levels of Use of AI Radiology

**Figure 1:**
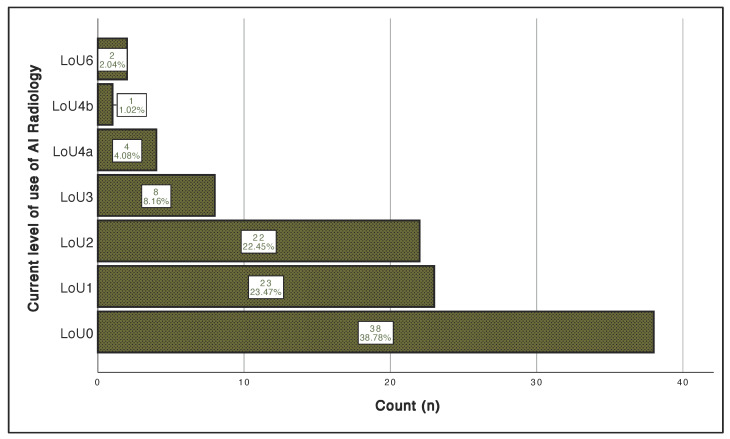
The current levels of use of AI radiology. The vertical axis represents the current level of use of AI. The horizontal axis represents the number of participants. LoU: level of use; AI: artificial intelligence.

Description of the level of AI use scale is presented in Appendix A, and the participants’ response is illustrated in Figure 1. Levels of AI use were very low, with 39% having no experience or significant knowledge (LoU0) and a few had mild experience (LoU3, 8%) or were using it in their routine practice (LoU4a-6, 7%).

### 3.4. Perceived Impact of AI on Radiology Workflow and the Radiology Profession

**Figure 2:**
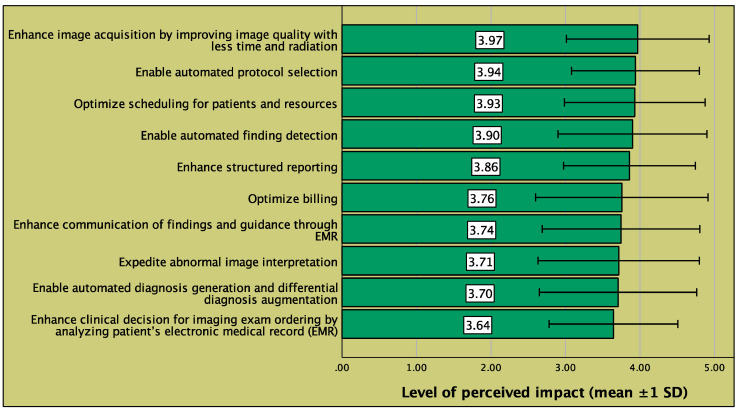
Perceived impact of AI on the different steps of standard radiology workflow. Bars represent the mean level of perceived impact, on a scale of 1 to 5 (1, no impact; 5, drastic impact), for AI on the given step of standard radiology workflow. EMR: electronic medical record; SD: standard deviation.

**Figure 3:**
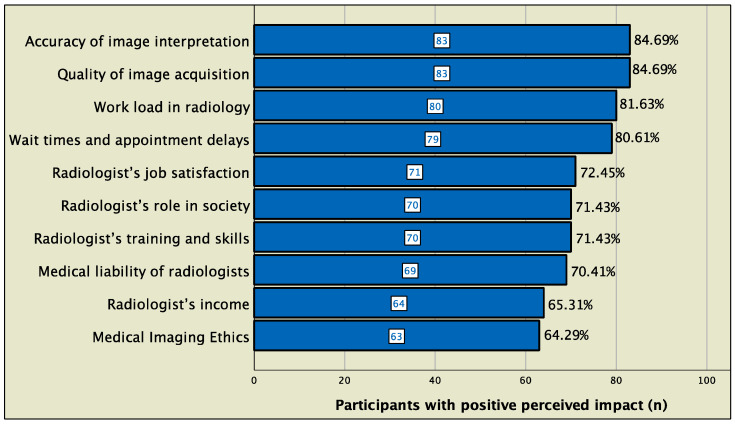
Attitudes regarding AI impact on the radiology profession. Bars represent the number of participants who perceived the impact of AI as being positive or very positive on the given aspect of the radiology profession.

Overall, the impact of AI radiology on radiology workflow was perceived to be high throughout the steps of radiology workflow, and the highest impact was perceived to be in enhancement of image acquisition (mean score, 3.97 out of 5), followed by enabling automated protocol selection (3.94 out of 5) and optimization of patient scheduling and resources (3.93 out of 5) (Figure 2). For the perceived impact on the radiology profession, a positive impact was most frequently perceived in technical and logistic aspects such as image interpretation (85%), image quality acquisition (85%), workload (82%), and wait times and appointment delay, whereas the perceived impact was relatively less positive in aspects related to prestige and regulation such as income (65%) and ethics (64%) (Figure 3). The perceived impact of AI radiology on workflow and profession is presented in further aspects in Figure 2 and Figure 3, respectively.

### 3.5. Internal Consistency of the Study Scales

All four study scales used in the present study showed high or very high reliability (Table 3). Consequently, scores were calculated for each scale, and the respective means and ranges are presented in Table 3 by reference to theoretical ranges.

### 3.6. Factors Associated with Perceived Impact of AI on Standard Radiology Workflow and on the Radiology Profession

Younger participants (age < 28 years) had a significantly higher perceived impact on both radiology workflow (mean ± SD score, 39.83 ± 8.00 versus 35.14 ± 8.29) and profession (mean ± SD score, 11.22 ± 5.95 versus 6.03 ± 8.57) compared to those aged 28 years and older respectively (*p* < 0.001). Participants with a postgraduate degree (Masters or PhD) also had a remarkably higher perceived impact of AI on both radiology workflow (mean ± SD score, 46.25 ± 6.18 versus 37.81 ± 8.30) and profession (mean ± SD score, 16.25 ± 7.50 versus 9.07 ± 7.28), compared to those with a bachelor’s degree alone (*p* < 0.05). Additionally, trainees with mixed academic/non-academic activity had a significantly higher perceived impact of AI on both the radiology workflow (mean ± SD score, 41.90 ± 5.31 versus 27.40 ± 9.11 and 33.96 ± 8.38) and profession (mean ± SD, 13.27 ± 4.41 versus 1.50 ± 2.88 and 3.82 ± 7.89) compared with those who had an exclusively academic or non-academic job activity, respectively (*p* < 0.001). Table 4 shows AI perceived impact scores in relation to demographic and professional factors.

### 3.7. Factors Associated with Perceived Ease of Use (PEoU) of AI

The PEoU score was higher among younger participants and those with mixed academic/non-academic activity compared with their counterparts (*p* < 0.001). Additionally, PEoU score was positively correlated with LoU of AI radiology (*R* = 0.41, *p* < 0.001), perceived impact on workflow (*R* = 0.62, *p* < 0.001), and perceived impact on the radiology profession (*R* = 0.70, *p* < 0.001). Among these significant factors, current professional activity (*B* = 0.55, *p* = 0.005), LoU (*B* = 0.15, *p* = 0.043), and perceived impact on profession score (*B* = 0.09, *p* < 0.001) were independently associated with the PEoU of AI in a multivariate model that explained 55.7% of the variance in PEoU score. Unadjusted and adjusted analyses examining factors associated with PEoU are tabulated in Table 5 and Table 6, respectively.

### 3.8. Factors Associated with Behavioral Intention (BI) to Use AI

The BI to use AI score was higher among younger participants and those having mixed academic/non-academic job activity (*p* < 0.001). It was linearly correlated with the LoU of AI radiology (*R* = 0.36, *p* < 0.001), perceived impact on workflow (*R* = 0.74, *p* < 0.001), and perceived impact on the radiology profession (*R* = 0.82, *p* < 0.001). The multivariate model showed that only perceived impact on workflow (*B* = 0.07, *p* < 0.001) and perceived impact on the profession (*B* = 0.16, *p* < 0.001) were independent factors for BI, explaining 71.2% of the variance in BI score. The results from unstandardized and standardized models of BI are presented in Table 5 and Table 6, respectively.

## 4. Discussion

### 4.1. Exposure and Levels of Use

The advent of AI is dramatically transforming healthcare in several aspects, with medical imaging being one of the most concerned branches. In a country such as Saudi Arabia, where the integration of data science and telehealth is being encouraged by the new vision of the government, the parallel investment in human capital is critical to the effectiveness of the new reforms [24,25]. We observed that radiology residents have low exposure to AI associated with inadequate knowledge, predicting low acceptance of AI in radiology. Additionally, less than 8% were practicing AI at their institution at variable levels. Conversely, the majority of the participants exhibited interest in learning AI. Exposure and knowledge about AI in radiology has been investigated in other local and international studies. A study by Khafaji et al. involving 154 radiology residents from the Saudi Board of Radiology showed that 42% of the participants reported being familiar with AI in medical imaging, while only 6.5% have taken courses in AI and ML and 4% had experience in AI [20]. Another national study by Alelyani et al. among 714 participants from different radiology-related positions, demonstrated that 61% of the participants were aware of AI in medical imaging; however, only 24% had previous or ongoing research activity on AI application in radiology [26]. By contrast, another study by Qurashi et al., which explored the perceptions towards AI implementation among 224 participants from different radiology-related positions, found that a majority (83%) declared being familiar with ML and AI concept. However, a minority have been exposed to or practiced AI (18%), deploring lack of training in formal curriculum [11]. Abuzaid et al. argued that radiographers in Saudi Arabia face challenges in acquiring AI-related education and training, and they reported a lack of education courses to facilitate AI use [15].

Internationally, a study from Singapore explored the level of familiarity as well as the interest and opinions of 125 radiologists from different diagnostic and interventional subspecialties. Among the participants, 15.2% considered themselves competent in AI in their radiology practice, 16.8% were actively involved in AI-related radiology research, and 19.2% had attended AI and data science courses during the last 5 years. On the other hand, the majority showed excitement towards learning AI [14]. Another study from the USA showed that only 23% of 95 interviewed thoracic radiologists had previous experience in AI [10].

### 4.2. Perceived Impact of AI on Radiology Workflow and Radiologist Profession

Radiology residents had high expectations about the usefulness of AI application throughout the steps of the radiology workflow. The roles endorsed ranged from improving image acquisition to assisting in clinical decisions. On the other hand, opinions were mixed regarding the impact AI would have on the radiologist profession. While more than 80% acknowledged a positive impact in assisting the radiologist in the technical and managemental aspects, 30–35% were concerned about the legal aspects and the effect of AI on the prestige of the profession. By considering the low levels of knowledge and exposure, these perceptions and attitudes have a great likelihood to be founded on preconceptions and myths. Hence, the perceived benefits or adverse effects of AI radiology implementation may be over- or underestimated by the participants. Enthusiasm about AI in radiology may be explained by the increasing demand, both quantitative and qualitative, in radiological services, resulting in daily workload and pressure over radiologists [27]. AI provides a great potential for enhancing the performance while reducing the workload thanks to computerized processing and DL functions [28,29]. However, the hidden part of the task is that AI decision-making automation requires a significant time for machine learning process, during which the clinical judgment and validation by a radiographer are required [28].

From the user’s (or eventual user’s) perspective, AI in radiology harbors a certain paradox where it would be undeniably beneficial for work efficiency while being detrimental for the future of the radiologist profession. Therefore, several studies have attempted to demystify the potential of AI in radiology workflow and, on the other hand, its presumed negative impact on the profession. In Saudi Arabia, Khafaji et al. reported a high percentage of radiology residents believed that AI would reduce the workload in radiology [20], while Qurashi reported that more than half of radiology personnel are concerned about the negative impact of AI on their profession [11]. A study from Australia and New Zealand indicated that the top perceived benefit of AI is to reduce time spent by specialists on monotonous tasks [30]. An internet-based Italian study that involved 1032 radiologists reported that two-thirds of the participants viewed AI as an aid to daily working practice, with remarkably positive attitudes regarding the effect of AI on improving accuracy and turnaround time in radiology. Yet, 60% of the participants were concerned about AI disrupting the radiologist’s professional reputation and 20% believed it will impact the income and recruitment opportunities in the profession [16]. By comparison, 35% of the participants in our study expressed concerns about income. In Singapore, 12% of radiologists were concerned about AI replacing human competency [14].

The development of AI in medical imaging has raised psychological, ethical, and legal concerns. An important psychological risk is related to the possibility that the poor explainability of most current AI systems and their lack of transparency could cause anxiety and distrust in patients and healthcare providers [31]. On the other hand, AI adoption might engender the propensity to favor an automated diagnosis over a diagnosis derived from scientific evidence and one’s expertise, and in the long term, clinicians might over rely on the machine-generated interpretation. Undoubtedly, patient health depends on the decision-making process, and in absence of specific regulations and policies, the use of AI system may lead to ethical, medico-legal, and liability issues [32].

The impact of AI on the radiology profession is still subject of debate. In particular, it was believed that it would be 5 years or less before AI had a noticeable impact on the profession [30]. A study from the USA reported that 32.7% and 64.3% of thoracic radiologists forecasted AI to have dramatic impact on the radiologist’s job by the next 10 and 20 years, respectively [10]. Conversely, a study conducted in the United States reported that none of the attending radiologists and only a very small proportion of the trainees believed that their jobs would be obsolete in the next 10–20 years [18]. In Saudi Arabia, radiology professionals believe that AI can potentially change the workflow in radiology with no effect on job security [33].

### 4.3. Acceptance of AI in Radiology 

Besides these concerns, misconceptions and negative attitudes are predictive for the cognitive and behavioral process leading to conditional acceptance and use of AI in clinical practice [22,23]. By using a theoretical framework based on the Technology Acceptance Model, we demonstrated that a positively perceived impact on the radiologist profession was correlated with greater PEoU and behavioral intention to use AI in radiology regardless how its perceived impact on the workflow is drastic. Hence, a negative perception is associated with low acceptance of AI radiology. The model developed in the present study supports that perceived impact of AI would explain 71.2% of the variance in behavioral intention to use it. This constitutes the major contribution of our study, demonstrating the importance of alleviating misconceptions and raising awareness about the positive impact of AI to enhance engagement among potential users. 

### 4.4. Limitations

The present study has two major methodological limitations. The first limitation is the small sample size, which is due the very low response rate. Thus, the statistical power for the achieved sample size (*N* = 98) for the unknown proportion (*p* = 50%) under the null hypothesis is 31.34%, which is low. The second limitation is the lack of stratification, which resulted in imbalanced distribution of the participants across regions and professional characteristics. Both limitations hinder the generalizability of the findings. Finally, the questionnaire had not been adequately validated. These limitations indicate that the study fits at best as a pilot study, which is potentially useful for providing the groundwork in future studies among radiology trainees in Saudi Arabia.

## 5. Conclusions

Levels of use of AI among radiology residents are very low. The perceived impact of artificial intelligence on radiology workflow and the profession is correlated to an increase in behavioral intention. It is crucial to enhance the theoretical and practical learning of AI among Saudi radiology trainees, to alleviate the misconceptions and enable efficient implementation of AI.

## Figures and Tables

**Table 1:** Participants’ demographic and professional characteristics (*n* = 98).

Parameter	Unit	Mean	SD
Age	years	27.59	2.02
**Parameter**	**Category**	**Frequency**	**Percentage**
Gender	Male	51	52.04
	Female	47	47.96
Province	Makkah	55	56.70
	Riyadh	17	17.53
	Eastern Province	16	16.49
	Madinah	4	4.12
	Jizan	3	3.09
	Aseer	2	2.06
Sector	Ministry of Health	77	78.57
	University	7	7.14
	Military	9	9.18
	Other	5	5.10
Academic degree	Bachelor’s	94	95.92
	Masters or PhD	4	4.08
Current professional activity	Academic	10	10.20
	Non-academic	28	28.57
	Mixed	60	61.22

Data are presented as (mean and SD) or (frequency and percentage), as indicated. SD: standard deviation.

**Table 2:** Exposure to and interest in artificial intelligence in radiology (*n* = 98).

Item	Levels, % (*n* = 98)				
**Familiarity**	**1 ○ Never heard about it**	**2 ○ Heard about it but not familiar with what it stands for**	**3 ○ Heard about it but barely understand what it is**	**4 ○ Familiar with its basics**	**5 ○ Have accurate knowledge about it**
AI	8.2%	15.3%	31.6%	42.9%	2.0%
ML	16.3%	15.3%	29.6%	36.7%	2.0%
DL	19.4%	19.4%	25.5%	33.7%	2.0%
Data science	16.3%	17.3%	25.5%	38.8%	2.0%
**Involvement**	**1 ○ No, and not interested**		**2 ○ No, but interested**	**3 ○ Yes**	
Reading journal articles about AI radiology	14.3%		46.9%	38.8%	
Attending AI radiology courses	15.3%		60.2%	24.5%	

All the data are presented as percentage. AI: artificial intelligence; ML: machine learning; DL: deep learning.

**Table 3:** Internal consistency of the four study scales.

Scale	No. Items	Cronbach’s Alpha	Internal Consistency Level	Score Statistics			Ref. Scale Range
				Mean	SD	Range	
Perceptions about AI impact on the radiology workflow	10	0.955	Very high	38.15	8.37	10, 50	10, 50
Attitudes regarding AI impact on the radiology profession	10	0.926	Very high	9.37	7.39	−9, 20	−20, +20
Perceived ease of use	3	0.883	High	11.88	2.00	9, 15	3, 15
Behavioural intention	3	0.888	High	12.21	2.00	8, 15	3, 15

AI: artificial intelligence; SD: standard deviation.

**Table 4:** Factors associated with perceived impact of AI on standard radiology workflow and on the radiology profession.

Parameter	Unit	Perceived Impact on Standard Radiology Workflow			Perceived Impact on the Radiology Profession		
		Mean	SD	*p*-Value	Mean	SD	*p*-Value
Age	<28 years	39.83	8.00	0.007 ^#^	11.22	5.95	<0.001 ^#^
	≥28 years	35.14	8.29		6.03	8.57	
Gender	Male	38.55	6.58	0.628 ^#^	9.02	6.60	0.630 ^#^
	Female	37.72	10.01		9.74	8.21	
Province	Makkah	38.51	8.87	0.433 *	10.11	7.47	0.592 *
	Riyadh	38.59	6.39		9.94	7.89	
	Eastern Province	35.44	8.97		6.19	7.73	
	Madinah	40.75	1.50		10.75	4.65	
	Jizan	34.67	12.86		8.67	7.09	
	Aseer	47.00	0.00		10.00	0.00	
Sector	Ministry of Health	39.30	6.98	0.057 *	10.05	7.29	0.214 *
	University	33.43	13.05		4.71	6.50	
	Military	32.89	12.44		9.00	7.98	
	Other	36.60	8.91		6.00	7.97	
Academic degree	Bachelor’s	37.81	8.30	0.048 ^#^	9.07	7.28	0.028 ^#^
	Masters or PhD	46.25	6.18		16.25	7.50	
Current professional activity	Academic	27.40	9.11	<0.001 *	1.50	2.88	<0.001 *
	Non-academic	33.96	8.38		3.82	7.89	
	Mixed	41.90	5.31		13.27	4.41	

All the data are presented as (mean and SD). *p*-values were calculated by * one-way ANOVA or ^#^ Student’s *t*-test. *p* < 0.05 was considered statistically significant. SD: standard deviation.

**Table 5:** Factors associated with perceived ease of use and behavioural intention to use AI.

Parameter	Unit	Perceived Ease of Use				Behavioural Intention			
		Mean	SD	*p*-Value		Mean	SD	*p*-Value	
Age	<28 years	13.16	1.35	<0.001 ^#^		12.76	1.64	<0.001 ^#^	
	≥28 years	12.17	1.34			11.23	2.22		
Gender	Male	12.78	1.29	0.875 ^#^		12.25	1.90	0.835 ^#^	
	Female	12.83	1.56			12.17	2.13		
Province	Makkah	12.78	1.42	0.977 *		12.45	2.04	0.820 *	
	Riyadh	13.06	1.39			12.12	1.96		
	Eastern Province	12.75	1.39			11.63	2.06		
	Madinah	12.50	2.52			12.25	2.06		
	Jizan	12.67	1.53			12.00	2.65		
	Aseer	13.00	0.00			12.00	0.00		
Sector	Ministry of Health	12.88	1.40	0.402 *		12.35	1.99	0.520 *	
	University	12.57	0.79			11.29	2.21		
	Military	12.11	1.96			11.78	2.22		
	Other	13.20	1.10			12.20	1.64		
Academic degree	Bachelor’s	12.77	1.42	0.176 ^#^		12.14	1.98	0.068 ^#^	
	Masters or PhD	13.75	1.26			14.00	2.00		
Current professional activity	Academic	11.40	1.51	<0.001 *		10.10	1.29	<0.001 *	
	Non-academic	11.79	1.17			11.18	2.29		
	Mixed	13.52	1.00			13.05	1.40		
**Score**		***B***	**95% CI**	***R***	***p*-Value**	***B***	**95% CI**	***R***	***p*-Value**
Level of use of AI radiology		0.40	0.22, 0.58	0.41	<0.001 ^†^	0.50	0.24, 0.75	0.36	<0.001 ^†^
Perceived impact on workflow		0.10	0.08, 0.13	0.62	<0.001 ^†^	0.18	0.14, 0.21	0.74	<0.001 ^†^
Perceived impact on profession		0.14	0.11, 0.16	0.70	<0.001 ^†^	0.22	0.19, 0.25	0.82	<0.001 ^†^

Data are presented as (mean and SD) or (unstandardized regression coefficient, Pearson’s correlation coefficient, and 95% confidence interval). *p*-values were calculated by * one-way ANOVA, ^#^ Student’s *t*-test, or ^†^ linear regression. *p* < 0.05 was considered statistically significant. *B*: unstandardized regression coefficient; 95% CI: 95% confidence interval; *R*: Pearson’s correlation coefficient; AI: artificial intelligence; SD: standard deviation.

**Table 6:** Independent factors associated with perceived ease of use and behavioural intention to use AI (stepwise linear regression).

Parameter	No. of Levels	Perceived Ease of Use ^#^				Behavioural Intention ^†^			
		*B*	95% CI		*p*-Value	*B*	95% CI		*p*-Value
Current professional activity	3	0.55	0.17	0.93	0.005 *	NI			
Level of use of AI radiology	(discrete)	0.15	0.01	0.29	0.043 *	NI			
Perceived impact on workflow	(discrete)	NI				0.07	0.03	0.11	<0.001 *
Perceived impact on profession	(discrete)	0.09	0.06	0.13	<0.001 *	0.16	0.12	0.21	<0.001 *
Model goodness-of-fit (*R*^2^)		0.557				0.712			

Data are presented as (standardized regression coefficient and 95% confidence interval). *p*-values were calculated by * stepwise linear regression. *p* < 0.05 was considered statistically significant. *B*: standardized regression coefficient; 95% CI: 95% confidence interval; AI: artificial intelligence; NI: variable not included in the model. ^#^ Factors removed from the model in the stepwise approach: age, gender, province, sector, academic degree, and perceived impact on workflow. ^†^ Factors removed from the model in the stepwise approach: age, gender, province, sector, academic degree, current professional activity, and level of use of AI radiology.

## Data Availability

Data generated during the study are available upon reasonable request.

## Appendix A

**Table A1:** Level of use of Artificial Intelligence in Radiology.

Level of Use of Artificial Intelligence Radiology	
**LoU0**	I have no experience in **AI Radiology**; I have no significant knowledge about it and I am doing nothing towards becoming involved in it
**LoU1**	I have acquired or am acquiring information about **AI Radiology**; I am exploring its value and its demands upon physicians and health institutions
**LoU2**	I think I am ready for **AI Radiology** implementation and am preparing for my first use
**LoU3**	I have already made my first steps in **AI Radiology**; I am using it superficially or whenever I need it
**LoU4a**	I am using **AI Radiology** in my routine practice but I have no idea about its impact on my patients or the quality of care
**LoU4b**	I am using **AI Radiology** and attempting to optimize my use to meet my patients’ needs and or improve my clinical practice
**LoU5**	I am using **AI Radiology** and coordinating my efforts with other colleagues and health professionals for best effect on patient care
**LoU6**	I am using **AI Radiology** and I think there are some necessary modifications to the system to achieve increased impact of patients; Or, I am using **AI Radiology** and I think its scope should be expanded to new goals

AI: artificial intelligence; LoU: level of use.

## Appendix B

**Table A2:** Perception of impact of artificial intelligence on radiology workflow.

To What Extent Do You Think Artificial Intelligence Can or Will Impact the Following Steps of Standard Radiology Workflow?						
1 ○ *No impact* 2 ○ *Small impact* 3 ○ *Moderate impact* 4 ○ *Large impact* 5 ○ *Drastic impact*						
01	Enhance clinical decision for imaging exam ordering by analyzing patient’s EMR	1 ○	2 ○	3 ○	4 ○	5 ○
02	Optimize scheduling for patients and resources	1 ○	2 ○	3 ○	4 ○	5 ○
03	Enable automated protocol selection	1 ○	2 ○	3 ○	4 ○	5 ○
04	Enhance image acquisition by improving image quality with less time and radiation	1 ○	2 ○	3 ○	4 ○	5 ○
05	Enable automated finding detection	1 ○	2 ○	3 ○	4 ○	5 ○
06	Enable automated diagnosis generation and differential diagnosis augmentation	1 ○	2 ○	3 ○	4 ○	5 ○
07	Expedite abnormal image interpretation	1 ○	2 ○	3 ○	4 ○	5 ○
08	Enhance structured reporting	1 ○	2 ○	3 ○	4 ○	5 ○
09	Enhance communication of findings and guidance through EMR	1 ○	2 ○	3 ○	4 ○	5 ○
10	Optimize billing	1 ○	2 ○	3 ○	4 ○	5 ○

EMR: electronic medical record.

## Appendix C

**Table A3:** Attitudes regarding Artificial Intelligence impact on the radiologist profession.

How Would the Implementation of Artificial Intelligence Impact Each of the Following Aspects of the Radiologist Profession?						
−2 ○ *Very negative impact* −1 ○ *Negative impact* 0 ○ *Mixed opinion, or no impact* +1 ○ *Positive impact* +2 ○ *Very positive impact*						
01	Medical Imaging Ethics	−2 ○	−1 ○	0 ○	+1 ○	+2 ○
02	Medical liability of radiologists	−2 ○	−1 ○	0 ○	+1 ○	+2 ○
03	Quality of image acquisition	−2 ○	−1 ○	0 ○	+1 ○	+2 ○
04	Accuracy of image interpretation	−2 ○	−1 ○	0 ○	+1 ○	+2 ○
05	Wait times and appointment delays	−2 ○	−1 ○	0 ○	+1 ○	+2 ○
06	Work load in radiology	−2 ○	−1 ○	0 ○	+1 ○	+2 ○
07	Radiologist’s role in society	−2 ○	−1 ○	0 ○	+1 ○	+2 ○
08	Radiologist’s income	−2 ○	−1 ○	0 ○	+1 ○	+2 ○
09	Radiologist’s training and skills	−2 ○	−1 ○	0 ○	+1 ○	+2 ○
10	Radiologist’s job satisfaction	−2 ○	−1 ○	0 ○	+1 ○	+2 ○

## Appendix D

**Table A4:** Perceived ease of use and behavioral intention of Artificial Intelligence use in routine radiology practice.

Please Rate Your Level of Agreement to the Following Statements:						
1 ○ *Extremely disagree* 2 ○ *Disagree* 3 ○ *I do not know* 4 ○ *Agree* 5 ○ *Extremely agree*						
**PEoU1**	Understanding the principles of **AI Radiology** would be easy for me	1 ○	2 ○	3 ○	4 ○	5 ○
**PEoU2**	Learning to operate **AI Radiology** would be easy for me	1 ○	2 ○	3 ○	4 ○	5 ○
**PEoU3**	I would find it easy to do all what I need to do in my practice using **AI Radiology**	1 ○	2 ○	3 ○	4 ○	5 ○
**BI1**	Assuming I have access to the system, I intend to use **AI Radiology**	1 ○	2 ○	3 ○	4 ○	5 ○
**BI2**	To the extent possible, I intend to use **AI** technology in all dimensions of my radiology practice	1 ○	2 ○	3 ○	4 ○	5 ○
**BI3**	I intend to encourage my colleagues to use **AI Radiology**	1 ○	2 ○	3 ○	4 ○	5 ○

PEoU: perceived ease of use; BI: behavioral intention; AI: artificial intelligence.
